# The Role of the Single Incision Laparoscopic Approach in Liver and Pancreatic Resectional Surgery

**DOI:** 10.1155/2016/1454026

**Published:** 2016-11-07

**Authors:** Nikolaos A. Chatzizacharias, Khaled Dajani, Jun Kit Koong, Asif Jah

**Affiliations:** ^1^Department of HPB and Transplant Surgery, Addenbrooke's Hospital, Cambridge University Hospitals NHS Foundation Trust, Cambridge, UK; ^2^Department of Surgery, Faculty of Medicine, University of Malaya, Kuala Lumpur, Malaysia

## Abstract

*Introduction*. Single incision laparoscopic surgery (SILS) has gained increasing support over the last few years. The aim of this narrative review is to analyse the published evidence on the use and potential benefits of SILS in hepatic and pancreatic resectional surgery for benign and malignant pathology.* Methods*. Pubmed and Embase databases were searched using the search terms “single incision laparoscopic”, “single port laparoscopic”, “liver surgery”, and “pancreas surgery”.* Results*. Twenty relevant manuscripts for liver and 9 for pancreatic SILS resections were identified. With regard to liver surgery, despite the lack of comparative studies with other minimal invasive techniques, outcomes have been acceptable when certain limitations are taken into account. For pancreatic resections, when compared to the conventional laparoscopic approach, SILS produced comparable results with regard to intra- and postoperative parameters, including length of hospitalisation and complications. Similarly, the results were comparable to robotic pancreatectomies, with the exception of the longer operative time reported with the robotic approach.* Discussion*. Despite the limitations, the published evidence supports that SILS is safe and feasible for liver and pancreatic resections when performed by experienced teams in the tertiary setting. However, no substantial benefit has been identified yet, especially compared to other minimal invasive techniques.

## 1. Introduction

Single incision laparoscopic surgery (SILS), first described by Inoue et al. [1] more than two decades ago for an appendectomy procedure, has gained support for the benefit of improved cosmesis compared to the multiport laparoscopic approach, as well as the potential reduction in the risk of port-related complications, such as bleeding and visceral injury, less postoperative pain, shorter length of stay, and quicker return to work [2–6]. This innovative approach has been further applied to a broad range of operations, such as cholecystectomy, gastrectomy, colectomy, and splenectomy [7–11]. With regard to liver and pancreas surgery, data on the use of SILS are still limited to case reports and small series.

The purpose of this narrative review is to analyse the published evidence on the use and potential benefits of SILS in hepatic and pancreatic resectional surgery for benign and malignant pathology.

## 2. Methods

A literature search of the Pubmed and Embase databases was performed by two independent researchers (NAC and KD) using the search terms “single incision laparoscopic”, “single port laparoscopic”, “liver surgery”, and “pancreas surgery”. The search was confined to English manuscripts. As this is a narrative review, ethical approval was not required. Relevant references cited in the literature were reviewed and included where appropriate.

## 3. Results

**Figure 1:**
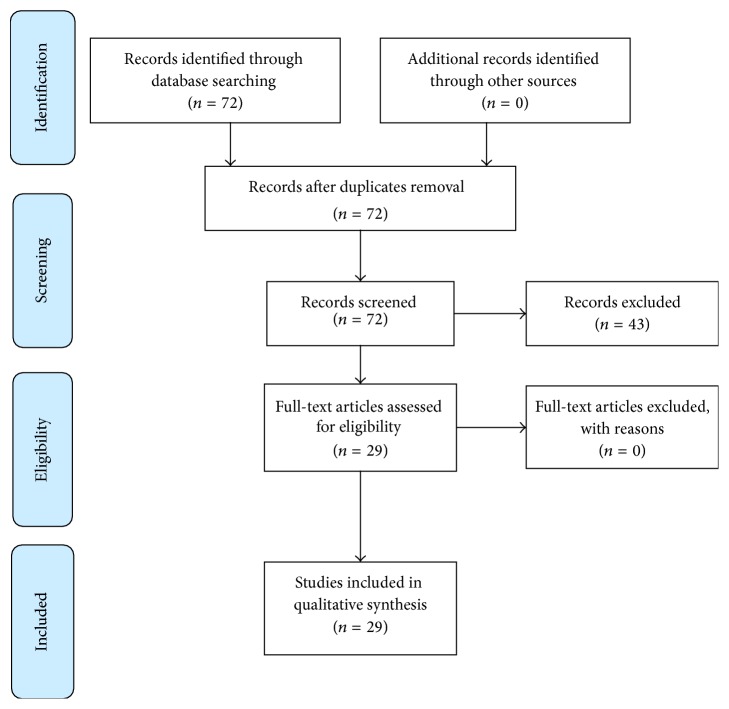


Initial literature search identified 51 publications for liver and 21 for pancreatic surgery. Cases of liver cysts deroofing were excluded from the analysis. Due to the limited amount of data, case reports were included. After review of publications, 20 manuscripts for liver and 9 for pancreatic SILS resectional surgery were deemed relevant and included in the analysis (Figure 1).

### 3.1. Liver Resectional Surgery

Over the last couple of decades, significant progress has been made in the field of minimally invasive liver surgery. It is now well established that laparoscopic liver resections are feasible and safe and produce comparable oncological outcomes to open resections, while resulting in shorter hospital stay and blood loss [40–42]. SILS is the most recent development in the laparoscopic approaches to liver surgery with increasing amount of data presented in the literature. Nonetheless, no studies comparing SILS with open or conventional multiport laparoscopic or robotic liver resectional surgery are currently available in the literature.

Various limitations have been described with the SILS approach, mainly with regard to the size and location of the lesions and the body mass index (BMI) of the patient. Easily accessible, superficial lesions in segments II, III, IV, V, and VIII [20, 28] are preferable, even though bigger or more technically challenging resections for less favourably located tumours have been described with increased experience in the technique (Table 1). With regard to the size of the lesions, most groups adopted a cut-off of <2.5–5 cm in diameter for malignant and <10 cm for benign tumours [12, 14, 20–22, 27, 28, 31]. Resection of larger malignant lesions has also been described [15, 20, 22]; however the potential extension of the incision for extraction of large specimen defeats the purpose of SILS [27]. Other contraindications include vascular or extrahepatic involvement and morbid obesity [20, 27, 28]. Even though a history of upper abdominal surgery is a relative contraindication for some groups [28], SILS liver resections in patients with previous hepatectomies [22], as well as a synchronous liver and colonic resection [22], have been described.

A detailed description of the technique is beyond the remit of this review. Briefly, the patient is positioned supine in reverse Trendelenburg, with the legs apart to facilitate the position of the primary surgeon [15, 21, 22, 26–28]. Patient positioning in left lateral or semilateral positions has also been described [15]. Transumbilical incision with a 3-trocar technique has been used by most groups, while right upper quadrant incision has also been described [15, 24]. The latter may become useful in the setting of portal hypertension with umbilical varices or lesions in distant segments. Standard liver resection techniques were used with a combination of ultrasonic and other energy devices, clips, and staplers. Articulating instruments and scopes were also used in some cases.

Median operative time was between 70 and 227 minutes (Table 1). For larger resections (right or left hepatectomy) reported operative times varied between 110 and 545 minutes. A 0–26% conversion rate to multiport laparoscopic or open procedure and acceptable blood loss for liver resectional surgery were reported (Table 1). Median length of stay was reported between 1 and 11 days (between 2 and 10 days for large resections), with longer length of stay being attributed to postoperative complications.

### 3.2. Pancreatic Resectional Surgery

As with liver surgery, minimally invasive approaches have been gaining support in the field of pancreatic surgery. Published evidence suggest that laparoscopic pancreatic resections have comparable oncological outcomes to open surgery and additional benefits with regard to postoperative pain and morbidity [43–45]. The SILS approach is less well established, with only a few cases and small series reported in the literature. Indications for SILS pancreatic resections included a variety of pathologies, benign and malignant (Table 2). Due to the technical challenge, strict selection criteria are usually used. Smaller lesions (<3.5 cm) are preferable, even though resections of larger ones have been described. Ideally, patients should have a low BMI, no history of previous abdominal surgery, and strong preference for cosmesis [38]. All published cases have been performed for favourably located lesions in the body and/or tail of the pancreas and include distal pancreatectomies, with or without splenic preservation. Exceptions are two local excisions of lesions in infants, with one of them being a case of enucleation of pancreatoblastoma from the head of the pancreas [39].

As with every laparoscopic procedure, patient positioning is of high importance. Supine [2, 32, 34, 36, 38, 39], right lateral [35], and semilateral [33, 37] positions with [2, 32, 34, 38, 39] or without legs apart have been described, while reverse Trendelenburg after establishing the pneumoperitoneum was used by all groups. The surgical technique described is similar among the reports with minor modifications. Umbilical incision was used mainly, with one group describing left pararectal incision for very distal lesions [33]. Most commonly a 3-trocar technique [32, 34, 35, 37–39] was used, while the use of scope and instruments varied (angulating and straight both reported). Subsequent dissection followed the standard laparoscopic steps with the use of an energy device for the sealing of smaller vessels, while the main splenic vessels were secured generally with the use of staplers or clips. Staplers were used for the pancreatic parenchymal transection. Gastric traction sutures have been described by some groups to facilitate better exposure of the pancreas [2, 32, 34, 35, 37]. Median operative time has been reported between 145 and 330 minutes with a 0–19% conversion rate and acceptable levels of blood loss (Table 2). Median length of stay varied between 2 and 7 days, while the most commonly reported complication was postoperative pancreatic fistula formation.

Despite the small number of cases, two single centre retrospective studies compared the results of the SILS approach to those of conventional laparoscopic distal pancreatectomy [33, 38]. Both reported no significant difference in the patients' characteristics between the two groups. More specifically, there was no significant difference with regard to patients' age [33, 38], ASA class [33], gender [38], and weight and BMI [33, 38]. Similarly, there was no significant difference in the size [33, 38] or type [38] of the lesions. Intraoperative parameters, such as operative time [33, 38], blood loss [33, 38], and conversion rate to open procedure [38], were also comparable, as well as postoperative parameters, such as pain [38], length of stay [33, 38], and complications [33, 38].

A comparison between SILS and robotic distal pancreatectomy and splenectomy has also been reported, based also on a retrospective analysis of a single centre cohort, which included cases performed for malignant disease [36]. With the exception of the longer operative time reported with the robotic approach (297 versus 254 minutes, *p* = 0.03), no significant differences were identified with regard to patients' age, BMI, blood loss, conversion rate, and size of the tumours. Of note, the group acknowledged the preference towards the SILS approach in patients with normal or low BMI, as the operating space in these patients may not be sufficient for the effective use of the robotic arms. Postoperative complication rate was also comparable between the two groups; nonetheless a case of mortality was reported in the robotic group.

## 4. Discussion

SILS is one of the latest evolutions in minimal invasive surgery and has been increasingly utilised in abdominal surgery. The evidence on its use in liver and pancreatic resectional surgery is scarce and limited to published case reports and small case series.

The main advantage of SILS is cosmesis, with the benefits of minimal or no-scar access advocated by various groups [14, 15, 27, 30, 34]. It also carries a lower risk for port site related complications, such as visceral injury and bleeding, as well as potentially less postoperative pain, reduced length of stay, and quicker return to work [2–6]. In liver and pancreatic resectional surgery, the benefits of SILS are still unclear. Due to the lack of prospective and randomised comparative studies between SILS and other minimally invasive approaches, such as the conventional multiport or robotic techniques, the evidence is currently based on retrospective analyses of small case series [33, 36, 38]. However, as SILS is a relatively new approach and the international experience is still small, the potential benefits might be more obvious in the future.

SILS is considered less invasive than standard multiport laparoscopy but has significant technical difficulties and limitations. The main one arises from difficult instrumentation due to the lack of space and triangulation. Therefore, SILS is mainly limited to low BMI patients with no history of previous abdominal surgery. Articulated laparoscopic telescopes and instruments have also been utilised in order to overcome this problem, but with significant increase in the cost of the operation [34]. In the context of major resectional surgery, such as liver and pancreatic surgery, the lack of space and triangulation might compromise the dissection and potentially the resection margins, while important manoeuvres, such as access to the hilum, Pringle's, or other emergency haemostatic manoeuvres, become very difficult to apply [27]. The length of the instruments also poses a potential problem for liver resections and also for small distal pancreatic lesions. With SILS through an umbilical incision, sometimes instruments are not long enough to reach the entire dissection surface. Some groups have described the use of right or left upper quadrant ports to overcome this problem [15, 24, 33] and even the design of customised longer instruments [15]. Furthermore, the small number of ports and space limit retraction capabilities. This is particularly important in the setting of pancreatic resectional surgery, where stomach traction sutures have been used by some groups [2, 32, 34, 35, 37].

Many surgeons prefer to leave an abdominal drain in the setting of liver and more commonly pancreatic resections. Some groups reported the use of the umbilical port as a drain exit site [2, 35], while the use of an additional 5 mm port that can subsequently be converted into the drain exit site has also been reported [34]. Although a potential disadvantage of SILS could be the increased risk for the development of incisional herniae due to the longer incision for the insertion of the SILS port system, this was not supported by the published evidence (Tables 1 and 2).

With regard to its main potential benefit, improved cosmesis, many reports suggest good to excellent results [13, 21, 26, 30, 34, 35, 38], with less scarring [38] and improved cosmesis [15, 28] for both liver [13, 15, 21, 26, 28, 30] and pancreatic resections [34, 35, 38]. Despite the fact that many series reported high levels of patient satisfaction [13, 14, 21, 28], only one study measured this during the first postoperative follow-up visit after SILS liver resection [21]. When asked to categorise their aesthetic satisfaction to poor, fair, good, or very good, the majority of patients (*n* = 4) were very satisfied, with one patient reporting good aesthetic result.

The vast majority of the experience with SILS in liver surgery refers to smaller resections (nonanatomical, left lateral sectionectomy and segmentectomies), with only 7 major hepatectomies reported in the literature (Table 1). The high level of technical difficulty limits, at least in the beginning of the learning curve, the use of the SILS approach to small, superficial, and easily accessible lesions. It is generally accepted that, ideally, patients should have a low BMI and no previous upper abdominal surgery, even though exceptions to these have also been reported [22]. Even though liver resections in cirrhotics may pose a greater challenge, SILS has also been described in this group of patients. Nonetheless, most cases were limited to early Child's A stage patients [12, 20, 28, 31], with only 3 cases reported in Child's B [12, 31] and 1 case in a Child's C patient [31]. The small number of reported SILS liver resections in the literature precludes direct comparison with laparoscopic surgery. Nonetheless, the median operative time of 70–227 minutes is not substantially different than the time (99–331 minutes) reported for laparoscopic hepatectomies [42]. Similarly, the estimated blood loss (<50–500 mL for SILS and 50–659 mLs for laparoscopic [42]) is also comparable. The wide range of the 0–26% conversion rate reflects the technical difficulties and long learning curve of the SILS approach. With increasing experience this is expected to approach the 4% conversion rate [42] of the laparoscopic approach. No mortality has been reported after SILS hepatectomy, while low complication rates and only 3 cases of liver specific complications were reported (Table 1). These results resemble the low mortality (0.3%) and morbidity (10.5%) rates after laparoscopic liver resections [42]. Keeping in mind the technical limitations of the SILS approach and despite the lack of randomised control trials and prospective comparative studies between SILS and multiport laparoscopic surgery, the published evidence generally supports the view that SILS is safe and feasible for liver resections when performed by experienced teams in the tertiary setting.

With regard to pancreatic surgery, only a limited number of reports is available with regard to SILS distal pancreatectomy with or without splenectomy, supporting its safety and feasibility in the appropriate setting. The vast majority of the cases were for benign disease with only 4 cases performed for malignant lesions (3 pancreatic ductal adenocarcinomas and 1 renal cancer metastasis to the pancreas) (Table 2). Any conclusions on the benefits of SILS for pancreatic resectional surgery should be made with caution, due to the lack of randomised trials and prospective studies. Based on two retrospective comparative case series, the results between SILS and the conventional multiport laparoscopic approach were comparable, without any substantial benefit in operative time, blood loss, postoperative pain, length of stay, and complication rate [33, 38]. This highlights the question of any real value of SILS in the context of pancreatic surgery. On the contrary, supporters of this approach would argue that its real benefits might become more obvious with increasing experience and evolving technology, an argument which was also valid in the early stages of development of laparoscopic surgery. Furthermore, this issue becomes more complicated after one retrospective case series reported comparable results between SILS and robotic surgery of the pancreas [36]. Once again, and in the absence of any strong evidence (prospective randomised trials), the value of SILS becomes questionable, especially as robotic pancreatic surgery has already gained wide acceptance in both benign and malignant resections. On the other hand, although no cost comparison has been published between the two techniques, the robotic approach is likely to have a higher capital cost.

In conclusion, published evidence has not shown any substantial benefit of SILS in the context of liver and pancreatic resectional surgery, especially compared to other minimal invasive techniques, such as multiport laparoscopic and robotic surgery. Further studies in the form of prospective and randomised controlled trials would be required to draw safe conclusions about the value of this innovative approach.

## Figures and Tables

**Table 1:** Published evidence on the use of SILS in liver resectional surgery.

	Procedure (number)	Indication (number)	Location of lesion (number)	Size of lesion (mm) (median and range) or (mean ± SD)	Conversion rate (%)	Operative time (min) (median and range) or (mean ± SD)	Blood loss (mL) (median and range) or (mean ± SD)	LOS (days) (median and range) or (mean ± SD)	Complications (number)
Claude et al., 2014 [12]	NALR (7)	FNA (1) Adenoma (1) HCC (2) CRCLM (2) HM (1)	Segment III (2) Segment V (2) Segment VI (3)	20 (20–47)	0	110 (60–150)	50 (25–150)	5 (1–13)	0

Tzanis et al., 2014 [13]	LLR (1) NALR (2)	Adenoma (1) HCC (1) FNH (1)	Segment II/III (1) Segment VI (1) Segment VI/VII (1)	38 (12–90)	0	110 (100–120)	<50 (<50–150)	3 (1–3)	1 (allergic reaction)

Wu et al., 2014 [14]	Proximal left (1) LLR (8) NALR (9)	HM (8) FNH (1) CRCLM (2) HCC (2) Hepatolithiasis (2) Adenoma (2)	Segment II (3) Segment IV (1) Segment V/VI (3) Segment VIII (2)	49 (20–105)	0	117.9 (55–185)	256.5 (30–830)	7 (3–10)	Pleural effusion (2) Wound infection (1) Incisional hernia (1)

Shetty et al., 2012 [15]	NALR (6) SLR (11) LH (1) LLR (4) RH (1)	HCC (23)	Segment I/VI (2) Segment II (5) Segment II/III (1) Segment IV (2) Segment V (1) Segment VI (11) Segment VII (1) Segment VI/VII (1)	36 (10–90)	26	205 (95–545)	500 (100–2500)	8 (5–16)	Bile leak (1)

Røsok and Edwin, 2011 [16]	NALR(1)	CRCLM (1)	Segment V (1)	15	0	—	120	5	Pulmonary oedema (1)

Toyama et al., 2013 [17]	NALR (1)	HCC/CCA (1)	Segment VI (1)	30	0	180	Minimal	—	None

Kobayashi et al., 2010 [18]	NALR (1)	HCC (1)	Segment III (1)	20	0	70	Minimal	1	None

Belli et al., 2011 [19]	NALR (1)	HCC (1)	Segment III (1)	—	0	130	Minimal	2	None

Pan et al., 2012 [20]	LLR (3) NALR (5)	HCC (4) Multiple cysts (2) CRCLM (1) HM (1)	Segment II/III (4) Segment IV (2) Segment VI (2)	47 (35–110)	0	89 (67–123)	58 (50–100)	3 (2–6)	None

Camps Lasa et al., 2014 [21]	LH (1) LLR (4)	CRCLM (4) Hydatid cyst (1)	Segment II (1) Segment III (2) Segment II/III (2)	24 (7–62)	0	135 (120–210)	—	3 (3-4)	None

Kim et al., 2014 [22]	LLR (1) NALR (2)	HCC (3)	Segment II (2) Segment III (1) Segment VI (1)	17 (10–36)	0	227 (142–228)	200 (200–250)	7 (3–8)	Pleural effusion (1)

Hu et al., 2011 [23]	LLR (1) NALR (1)	HM (2)	Segment II/III (1) Segment IV (1)	48 (15–80)	0	120	100	3	None

Gaujoux et al., 2011 [24]	LH (3) NALR (1)	HCC (1) Ovarian cancer metastasis (1) CRCLM (2)	Segment III (1) Segment IV (1) Segment III/IV (2)	14 (13–20)	0	115 (55–140)	38 (20–50)	2	None

Kim et al., 2013 [25]	LLR (1)	CRCLM (1)	Segments II/III (1)	80	0	315 (synchronous high anterior resection)	—	11	Atelectasis (1)

Machado et al., 2014 [26]	LLR (8)	Adenoma (6) FNH (1) CCA (1)	—	—	0	68 (45–100)	<100	1 (1-2)	None

Dapri et al., 2012 [27]	LLR (1) NALR (2)	Hydatid (2) CRCLM (1)	Segment II/III (1) Segment VII (1) Segment VIII (1)	—	0	158 (114–185)	350 (200–500)	5 (4-5)	None

Zhao et al., 2011 [28]	LLR (4) NALR (8)	HCC (2) Haemangioma (6) FNH (3) Adenoma (1)	Segment II/III (3) Segment III (5) Segment IV (1) Segment IV/V (1) Segment V (1) Segment V/VIII (1)	44 ± 26 (11–96)	17	80.4 ± 38.3 (35–160)	45 (20–800)	4 ± 1 (2–8)	Massive haemorrhage (1) Bile leak (1)

Cai et al., 2010 [29]	NALR (1)	HM (1)	Segment II/III (1)	38	0	75	80	2	None

Aldrighetti et al., 2011 [30]	LLR (1)	CRCLM (1)	Segment II/III (1)	35	0	145	50	4	None

Aikawa et al., 2012 [31]	NALR (8)	HCC (5) HM (1) Metastasis (1) NET (1)	Segment II (1) Segment III (2) Segment IV (2) Segment V (1) Segment VIII (2)	15 (9–30)	0	148 (141–235)	2 (0–10)	6 (3–11)	None

NALR: nonanatomical liver resection, LLR: left lateral resection, LH: left hepatectomy, RH: right hepatectomy, SLR: segmental liver resection, CRCLM: colorectal cancer liver metastasis, HCC: hepatocellular carcinoma, FNH: focal nodular hyperplasia, HM: haemangioma, CCA: cholangiocarcinoma.

**Table 2:** Published evidence on the use of SILS in pancreatic resectional surgery.

	Procedure (number)	Indication (number)	Size of lesion (mm) (median and range) or (mean ± SD)	Conversion rate (%)	Operative time (min) (median and range) or (mean ± SD)	Blood loss (mL) (median and range) or (mean ± SD)	LOS (days) (median and range) or (mean ± SD)	Complications (number)
Barbaros et al., 2010 [2]	DP+S (1)	RCC metastases (2)	23 (15–30)	0	330	100	7	POPF grade A (1)

Chang et al., 2012 [32]	DP (1)	SCN (1)	35	0	233	<100	3	None

Haugvik et al., 2013 [33]	DP (5) DP+S (3)	5 NET (5) SCN (1) IPMN (1) Fibrosis (1)	21 (10–45)	0	145 (98–223)	225 (30–400)	6 (3–15)	Port site infection (1) Port site bleeding (1) POPF grade B (2)

Machado et al., 2015 [34]	DP (18) DP+S (2)	NET (11) IPMN (6) MCN (3)	31 (9–70)	0	176 (110–340)	<50 (<50–250)	2 (1–5)	POPF grade A (4)

Misawa et al., 2012 [35]	DP (1)	Cystadenoma (1)	50 (35–65)	0	240	0	7	None
	DP+S (1)	MCN (1)		0	225	100	5	None

Ryan et al., 2015 [36]	DP+S (16)	PDA (3) NET (2) IPMN (2) SCN (2) MCN (1) Splenunculi (1) Splenic HM (1)	38 ± 30 (8–117)	19	190 (197 ± 40.7)	150 (246 ± 263.9)	4 (6 ± 3.8)	AF (1) Pneumonia (1) Colonic abscess (1)

Srikanth et al., 2013 [37]	DP+S (1)	NET (1)	35	0	—	—	5	Collection (1)

Yao et al., 2014 [38]	DP+S (7) DP (7)	MCN (6) SCN (3) Pancreatic cysts (3) Splenic artery aneurysm (2)	43 ± 22 (12–110)	7	166 ± 55	157 ± 162	7 ± 1 (5–10)	POPF grade A/B (1)

Zhang et al., 2015 [39]	Local excision (2) DP (1)	Pancreatoblastoma (1) Nesidioblastosis (2)	—	0	153 (120–200)	Minimal	6-7	None

DP: distal pancreatectomy, DP+S: distal pancreatectomy and splenectomy, NET: neuroendocrine tumour, IPMN: intraductal papillary mucinous neoplasm, SCN: serous cystic neoplasm, MCN, mucinous cystic neoplasm, PDA: pancreatic ductal adenocarcinoma, HM: haemangioma, AF: atrial fibrillation, POPF: postoperative pancreatic fistula.

## Abbreviations

SILS: Single incision laparoscopic surgery
BMI: Body mass index.
